# Complete Genome Sequence of an Avian Polyomavirus Strain First Isolated from a Pigeon in China

**DOI:** 10.1128/MRA.01490-18

**Published:** 2019-03-07

**Authors:** Qiuchen Li, Kai Niu, Haojie Sun, Yingju Xia, Shijing Sun, Jie Li, Fang Wang, Yu Feng, Xiaowei Peng, Liangquan Zhu, Xuezheng Fan, Yuming Qin, Jiabo Ding, Hui Jiang, Guanlong Xu

**Affiliations:** a National Reference Laboratory for Animal Brucellosis, China Institute of Veterinary Drug Control, Beijing, China; b College of Veterinary Medicine, Shandong Agricultural University, Tai’an, Shandong, China; c Department of Population Medicine and Diagnostic Sciences, College of Veterinary Medicine, Cornell University, Ithaca, New York, USA; University of Maryland School of Medicine

## Abstract

Avian polyomavirus can infect multiple bird species and cause inflammatory disease with high mortality in young psittacine birds. In this study, we sequenced and analyzed an avian polyomavirus isolated from a pigeon in China, strain APV-P, which is closely related to a polyomavirus in psittacine birds.

## ANNOUNCEMENT

Avian polyomavirus is a nonenveloped icosahedral capsid virus and has a circular double-stranded DNA (dsDNA) genome of approximately 5,000 bp in length. Avian polyomaviruses belong to the family *Polyomaviridae* (1). Polyomaviruses have a wide range of hosts, including horses (2), birds (3, 4), bats (5), and humans (6, 7). Avian polyomavirus usually causes inflammatory disease in birds and is associated with acute death, abdominal distention, and a feather abnormality in birds of the parrot family, especially budgerigars.

In 2018, 10 pigeon fecal samples were collected from a pigeon breeding facility for laboratory diagnosis; mild clinical signs (e.g., loss of feathers) were observed in these pigeons. Total DNA was extracted from the clinical samples with a QIAamp minikit (Qiagen, Hilden, Germany). The DNA extracted from clinical samples was used to amplify avian polyomavirus by PCR using the primers VP1 forward CAGTTCGAATTCATGTCCCAAAAAGGAAAAGG and VP1 reverse ACAGTGTCGACTTAGCGGGGAGCTTTGG to amplify the VP1-encoding region (1,000 bp). The PCR products were sequenced by using Sanger sequencing in both directions. All of the clinical samples were positive for the identical sequence; this indicated that these birds were infected with the avian polyomavirus (strain APV-P). To isolate the virus, the positive samples were filtered through a 0.22-μm filter and inoculated into chicken embryo fibroblast (CEF) cells in Dulbecco's modified Eagle medium (DMEM) supplemented with 2% fetal bovine serum. The cytopathic effect (CPE) was observed at 5 days postinfection. For further confirmation, DNA extracted from the samples was directly amplified by two pairs of primers to cover the full genome, 3.5-kb forward primer CGGGGGAGGCTTTACTATTTGTGG and 3.5-kb reverse primer AGGGGTAGGCGAGTTAGGCTGTGA and 3.4-kb forward primer CTTTTCCTCATCCCCTCCTTTGTC and 3.4-kb reverse primer CGCGCCCGTACTTTGGTTA. Two resulting PCR products (3.5 kb and 3.4 kb), each with a 2-kb overlap region, were sequenced using Sanger sequencing in both directions. The final genome was assembled with Lasergene software version 7.0 (DNAStar, Madison, WI, USA) (8).

The genome of the APV-P isolate was found to be circular and 4,981 bp in length, with 49% GC content. The genome structure of the APV-P isolate is similar to those of avian polyomaviruses and contains a noncoding control region (NCCR) and early and late regions. The early region encodes the large (599 amino acids [aa]) and small (145 aa) T antigens. The late region encodes the five proteins VP1 (343 aa), VP2 (341 aa), VP3 (235 aa), VP4 (176 aa), and VP4Delta (112 aa). The start codon for the *VP3* open reading frame (ORF) is located within the *VP2* ORF, and the amino acid sequence of VP3 is identical to the sequence of the C terminus (235 aa) of VP2. The VP4Delta protein shares the same amino acid sequence with VP4 except it is truncated between amino acids 68 and 131.

To characterize the genetic features of this APV-P isolate, the amino acid sequence of the APV-P isolate was compared with those of reference strains. The APV-P strain showed 99% identity with an avian polyomavirus isolated from psittacine birds, suggesting that it might be derived from an avian polyomavirus circulating in psittacine birds. In this study, we identified an avian polyomavirus in pigeon for the first time, which suggests that avian polyomaviruses have a wide host range and have the potential to threaten many different bird species.

### Data availability.

The complete genome sequence reported here was submitted to GenBank under the accession number MK061528.
